# Stroke Recovery Is a Journey: Prediction and Potentials of Motor Recovery after a Stroke from a Practical Perspective

**DOI:** 10.3390/life13102061

**Published:** 2023-10-15

**Authors:** Sheng Li

**Affiliations:** 1 Department of Physical Medicine and Rehabilitation, McGovern Medical School, University of Texas Health Science Center—Houston, Houston, TX 77025, USA; sheng.li@uth.tmc.edu; 2 TIRR Memorial Hermann Hospital, Houston, TX 77030, USA

**Keywords:** stroke, motor recovery, proportional recovery, spasticity, ICF

## Abstract

Stroke recovery is a journey. Stroke survivors can face many consequences that may last the rest of their lives. Assessment of initial impairments allows reasonable prediction of biological spontaneous recovery at 3 to 6 months for a majority of survivors. In real-world clinical practice, stroke survivors continue to improve their motor function beyond the spontaneous recovery period, but management plans for maximal recovery are not well understood. A model within the international classification of functioning (ICF) theoretical framework is proposed to systematically identify opportunities and potential barriers to maximize and realize the potentials of functional recovery from the acute to chronic stages and to maintain their function in the chronic stages. Health conditions of individuals, medical and neurological complications can be optimized under the care of specialized physicians. This permits stroke survivors to participate in various therapeutic interventions. Sufficient doses of appropriate interventions at the right time is critical for stroke motor rehabilitation. It is important to highlight that combining interventions is likely to yield better clinical outcomes. Caregivers, including family members, can assist and facilitate targeted therapeutic exercises for these individuals and can help stroke survivors comply with medical plans (medications, visits), and provide emotional support. With health optimization, comprehensive rehabilitation, support from family and caregivers and a commitment to a healthy lifestyle, many stroke survivors can overcome barriers and achieve potentials of maximum recovery and maintain their motor function in chronic stages. This ICF recovery model is likely to provide a guidance through the journey to best achieve stroke recovery potentials.

## 1. Introduction

Strokes are a leading cause of adult disability [1]. There are approximately a total of 7 million stroke survivors in the U.S [2], and about 133 million worldwide [1]. Stroke survivors can face consequences that may last the rest of their lives. These consequences include impairments related to thinking or memory, movement, sensation (e.g., vision or hearing), verbal, swallowing, or emotional functioning (e.g., personality changes, depression). These impairments not only affect individuals, but can also have lasting effects on families, caregivers and communities. Among the important goals of stroke rehabilitation are to improve body function and to maximize functional independence, participation and social reintegration, via coordinated delivery of therapies and interventions in an interdisciplinary approach.

## 2. Prediction of Motor Recovery

More than 80% of hospitalized patients after a stroke have some degrees of hemiparesis [3]. Hemiparesis includes negative symptoms, such as weakness and loss of dexterity, and positive symptoms, such as spasticity and abnormal synergy. Stroke survivors recover from hemiparesis spontaneously, but only to a certain degree. Spontaneous motor recovery occurs mainly in the first 3 to 6 months post stroke [4,5]. Prediction of who will recover after a stroke and to what extent has been a constant focus for researchers, clinicians, patients and family members in the field of rehabilitation. Motor impairment and recovery are often assessed and tracked by the Fugl-Meyer Motor Assessment (FMA) scale [6]. Prabhakaran et al. [7] proposed a proportional recovery rule. According to this rule, the majority of stroke survivors are expected to recover approximately 70% of their maximum potentials at 3 months after a stroke. For the upper limb, the maximal potential recovery is the difference between the maximal possible FMA score (66) and the initial FMA score (FMA_initial_) within the first week after the stroke. The predicted amount of motor recovery (Δ in FMA) equals 0.7 × (66—FMA_initial_). This rule has been replicated in many studies on upper-limb recovery [8], but limited to those with mild to moderate motor impairments, i.e., fitters. The non-fitters whose recovery does not follow the proportional recovery rule often have severe motor impairments, i.e., a very low initial FMA score. This proportional recovery rule was also observed in recovery of lower limb motor impairments [9], as well as in other domains, including somatosensory impairment [10], spatial-visual neglect [11,12] and aphasia after a stroke [12]. These consistent observations on recovery in different domains indicate that there exists a general extent of spontaneous recovery in the first three months for a subgroup of stroke survivors. However, the proportional recovery rule has been challenged due to various confounds, namely mathematical coupling and statistical bias [13], or the ceiling effects of FMA particularly in those with mild motor impairment [14].

These clinical observations of proportional recovery are accompanied by relevant parallel neurophysiological measures. Byblow et al. found that, in a cohort of 93 first-ever ischemic stroke survivors, those with the presence of motor evoked potentials (MEP+) from the paretic wrist extensors 5 days after a stroke recovered approximately 70% of the maximum recovery possible by 12 weeks; similarly, the ipsilesional resting motor threshold was also resolved by 70%, while MEP- survivors did not demonstrate proportional recovery [15]. Since the presence of MEP reflects integrity of the corticospinal tract, these results provide physiological support for this rule of proportional recovery in those fitters, at least at the population level. To better predict motor recovery for individuals, other confounding factors are included in a later prediction algorithm (PREP2), such as age; the presence or absence of the upper-limb motor evoked potentials elicited with transcranial magnetic stimulation; and the stroke lesion load obtained from MRI or stroke severity assessed with the NIHSS score [16]. The algorithm makes correct predictions of upper-limb functional outcomes at 3 months after a stroke, however, only for 75% of patients [16]. In these observational studies [7,8,15,16], the proportional recovery rule appears to reflect spontaneous biological recovery and predict the extent of motor recovery for the majority (about 70%) of stroke survivors.

The proportional rule offers valuable information to provide a reasonable prediction of spontaneous recovery for majority of stroke patients in the early recovery phase. For some patients with severe impairments, spontaneous recovery may take longer and the initial assessment may not accurately reflect their recovery potentials. For example, 6 out of 11 initially MEP- subjects later recovered MEPs at varying times with various levels of recovery [15]. In the early spontaneous recovery period, CNS is highly plastic and sensitive to interventions [17]. With neuromodulation and motor training, MEP- patients can achieve meaningful functional gains [18].

## 3. Functional Recovery and an ICF Model of Stroke Recovery

**Figure 1:**
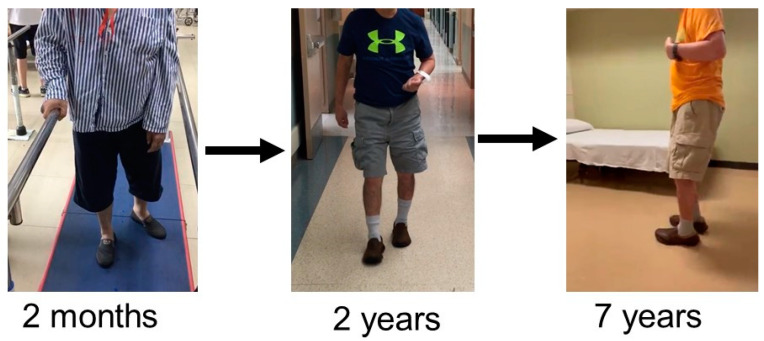
A gentleman had a middle cerebral artery ischemic stroke and resultant left hemiparesis at the age of 60. He was able to walk 2 months post-stroke, with support from parallel bars on his right side. As his recovery progressed, spasticity emerged and developed in his left arm and leg muscles, i.e., spastic hemiplegia in the chronic phase. His gait continued to improve and he walked with a cane. His left arm was in a flexed posture and did not swing. His knee was in mild flexion during the stance phase, and his ankle was plantarflexed during the swing phase. At 2 years post-stroke, he was able to walk at a moderate speed with legs alternating during walking. His hip flexor spasticity progressively worsened. At 7 years post-stroke, he was still able to walk, but at a very slow speed. His right leg was not able to advance and pass the left foot because of his left hip flexor spasticity, i.e., step-to gait [26].

In real-world clinical practice, stroke survivors continue to improve their motor function beyond the spontaneous recovery period, though at a lower rate in the subacute and chronic stages [19,20,21]. For example, a person with left hemiparesis continues to improve his walking from walking with parallel bars, to walking with a cane, and then walking without assistance at a moderate speed at 2 years post stroke, although he still walks in an abnormal pattern with compensatory mechanisms (Figure 1). According to the International Classification of Functioning, Disability and Health (ICF), improvement in walking performance is considered “recovery”, i.e., functional recovery [22]. However, the development of complications, such as spasticity [23] and sarcopenia [24,25], is likely to interfere with motor function in the later phase of motor recovery, as shown in Figure 1.

**Figure 2:**
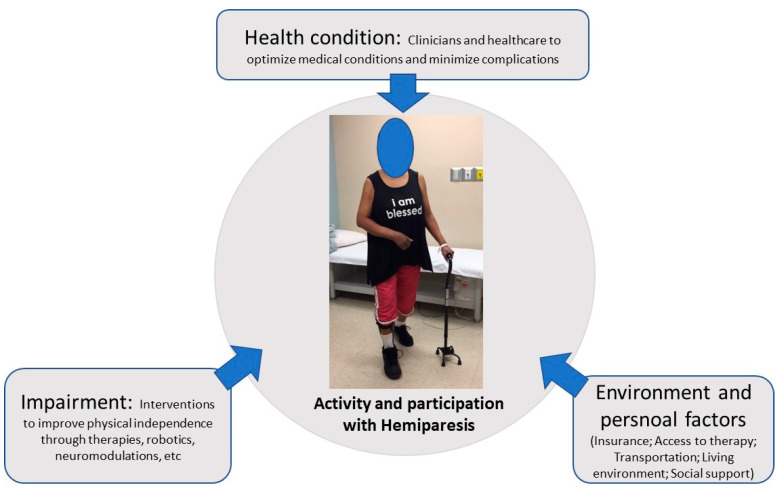
An ICF model of stroke recovery. See text for details.

Conceivably, in addition to factors that are important for spontaneous recovery [16], the extent and duration of continuous functional recovery depend on a number of other factors, such as management of complications and comorbidities, access to and participation in rehabilitation, and family and societal support. However, management approaches for maximum recovery are not well understood and articulated. Within the ICF theoretical framework, optimal recovery and independence of an individual with hemiparesis could be achieved through a combination of optimization of medical and neurological conditions, effective therapeutic interventions and assistive devices, and strong environmental and family support. Accordingly, an ICF recovery model is proposed to understand the ways to optimize the potentials of stroke recovery (Figure 2). This model allows a systematic approach to identify opportunities and manage potential barriers to maximize and realize the potentials of functional recovery. These factors are elaborated in details in the following sections.

### 3.1. Optimization of Health Conditions (Medical and Neurological)

There is well-established evidence that a dedicated stroke unit and two critically important inventions—intravenous thrombolytic drug treatment and endovascular mechanical thrombectomy—can significantly impact a patient’s clinical outcome at stroke onset and ultimately the recovery course. Risks of medical and neurological complications are high in the early recovery phase. Immediately after a stroke, a neuroinflammatory process starts in the brain, triggering a systemic immunodepression mainly through excessive activation of the autonomous nervous system [27]. Stroke patients are susceptible to infections. The most common infections are pneumonia and urinary tract infection; both occur in ≈10% of ischemic patients [28] and ≈40% in hemorrhagic patients [29]. Experimental and clinical data suggest that systemic infections enhance autoreactive immune responses against brain antigens and thus negatively affect outcomes [28]. Pneumonia increases unfavorable outcomes and mortality in patients with strokes. Seizures are frequently seen after cerebrovascular accidents. About 6% of cases developed early seizures (within 1 week) [30,31]. Acute symptomatic or early seizures affect between 3% and 6% of all stroke patients [32]. In addition to infection and seizure, intracranial hemorrhage, recurrent ischemic stroke and ischemic heart disease are the most common causes of acute care transfer among stroke inpatients, thus interrupting stroke rehabilitation [33]. Other conditions may interrupt the rehabilitation process temporarily, such as pulmonary embolus and deep vein thrombosis (DVT). The overall incidence of DVT after an acute stroke within two weeks was 14.4% [34].

Some complications can limit their participation in therapy, for example pain and depression. Stroke-related pain is present in 21% of stroke survivors and is associated with sensorimotor impairments and depression [35]. The complex regional pain syndrome (CRPS) that occurs after a stroke is often called shoulder–hand syndrome. Its prevalence ranges from 12.5% to 50% [36,37,38]. Post-stroke pain is often insufficiently recognized or inadequately treated. Patients’ activities of daily living and participation in therapy are negatively affected [39]. It has been shown that the activity status of the affected upper limb was negatively associated with the pain intensity in patients with post-stroke CRPS [40]. Post-stroke depression (PSD) is recognized as the most common neuropsychiatric complication following a stroke. Its symptoms develop within three to six months after a stroke event and affect 20–65% of stroke patients [41]. Depression is associated with less engagement in therapy and a worse functional outcome [42]. Vision impairment is prevalent and persistent (up to 93% of stroke survivors). This negatively affects their participation and engagement in therapy [43,44]. Cognitive impairment is a frequent consequence of strokes [45] and impacts patient engagement during inpatient stroke rehabilitation as well [46].

Nutritional support plays a critical role in health optimization for stroke recovery. In the acute phase, the risk of malnutrition is high. Malnutrition was identified in 25.8% of patients in a recent multicenter prospective study of 2962 acute stroke patients without pre-stroke disability [47]). Malnutrition is likely attributable to oropharyngeal dysphagia at this stage, which is found in up to 45% of stroke patients, using bedside screening techniques [48]. Delay in early screening for swallowing capacity in acute stroke patients is detrimental to outcomes, possibly due to delaying nutritional provision or through inappropriate feeding leading to aspiration. Compared to those who received swallow screening within 4 h of admission, a delay between 4 and 72 h was associated with greater risks of pneumonia, prolonged length of stay in a hyperacute stroke unit, and even mortality rate [49]. Although dysphagia itself was not a significant predictor of any of the outcomes measured, overall functional dependency was the most significant predictor of poor oral fluid intake and fluid-related adverse health outcomes in sub-acute strokes [50].

Taken together, stroke patients may have an initial poor functional assessment due to acute events and complications. However, after successful treatments and health optimization, more than 40% recover to good outcomes within 1 year [51]. These factors could account for a good recovery of non-fitters in the proportional recovery observational studies, and they should be taken into account for prediction of motor recovery. It is thus prudent to avoid early pessimistic prognostication until after successful treatment of these complications.

### 3.2. Effective Therapeutic Interventions

A comprehensive rehabilitation program is essential for stroke recovery. A standard motor rehabilitation program involves physical therapy and occupational therapy, which are usually delivered with assistive devices and technologies. The effectiveness of therapeutic interventions with pharmacological agents and advanced technologies and devices has been extensively explored. Optimal interventions, including content and modality, dosing, timing and combinations of these are still under investigation.

#### 3.2.1. Pharmacological Interventions

Numerous experiments have studied pharmacological interventions for motor recovery. Selective serotonin reuptake inhibitors (SSRI) are the most studied medication, including the FLAME [52], FOCUS [53], AFFINITY [54] and EFFECTS [55] trials. Although the initial FLAME trial showed promising results, a meta-analysis of 76 studies with 13,029 participants has revealed high-quality evidence that SSRIs alone do not make a difference to disability or independence after strokes as compared to a placebo or usual care [56]. Similarly, after promising results of dopamine agents on enhancing motor recovery when given in combination with physical therapy from 53 stroke participants [57], a large DARS trial with 1574 participants reported that co-careldopa in combination with routine therapies did not improve walking after strokes [58]. Other medications, such as D-amphentamine, Niacin, inosine and citicoline all showed some positive results in studies with small samples, but not in studies with large samples [59]. Many cofounding factors are discussed, such as stroke lesions, subject selections and outcome assessments. Pharmacological interventions benefit a subgroup of stroke survivors, e.g., SSRIs are given to patients with depression to improve their participation in therapy thus to facilitate motor recovery. In other words, stroke recovery is likely to be improved by precision medicine.

On the other hand, it is of the same importance to avoid medications that could negatively affect stroke survivors. It has been shown that anticholinergics and sedatives are independent factors associated with the time to recovery of activities of daily living and postural balance [60].

#### 3.2.2. Timing of Physical Interventions and Modalities

Spontaneous biological recovery is mostly attributable to a time-limited period of neuroplasticity [61]. To maximize the effectiveness of rehabilitative therapies after stroke, it is critical to determine when the brain is most responsive (i.e., plastic) to sensorimotor intervention and to focus such efforts within this period. In a recent clinical trial, Dromerick et al. compared an additional 15 to 20 h of upper-limb motor intervention given after fewer than 30 days (acute), 2 to 3 months (subacute) or greater than 6 months (chronic) with standard care (control). The outcome (the Action Research Arm Test, ARAT) was assessed over a year after the stroke. The results showed that only the subacute group surpassed the minimum clinically important difference on ARAT in comparison with the control group (ARAT = +6.87 ± 2.63 points). The acute group showed significant but smaller improvement (+5.25 ± 2.59 points). The chronic group showed no significant improvement compared with controls (+2.41 ± 2.25 point). This trial demonstrated that an early sensitive window exists, consistent with the first 2 to 3 months after the stroke. Additional therapy interventions during this critical period may change the recovery trajectory [17]. The optimal intervention dose and content to deliver within the sensitive window is an important question for future research.

#### 3.2.3. Optimizing the Intervention Dose

Motor recovery requires repetitions. Intensive and repetitive task-specific training of motor tasks and activities is recommended after a stroke [62,63]. In the acute rehabilitation phase (within 3 weeks post-stroke), robotic-assisted gait training (RAGT) is feasible to provide a higher dose of task-specific gait training for non-ambulatory stroke survivors. As compared to the conventional group (527 steps/day), the RAGT group had a much higher number of steps (1870 steps/day) and greater motor gain (32.3 vs. 17.9) at discharge [64]. When given in the subacute period, stroke survivors had clinically meaningful improvement after 20 h of additional therapy [17]. In the chronic phase, Ward et al. reported significant improvement in the upper-limb function in stroke survivors after a total of 90 h of therapy over 3 weeks (6 h per day), and the improvement was maintained at 6 months after the intervention [65]. In another study, chronic stroke survivors with moderate to severe motor impairments had significant improvement in their upper-limb function after 150 h of training and continued to improve after 300 h of training (5 h/day, 5 days/week for 12 weeks). The improvement was sustained for at least 3 months [66]. According to a recent Cochrane review, there is currently insufficient evidence to recommend a minimum beneficial daily amount in clinical practice. However, if the increase in time spent in rehabilitation exceeds a threshold, this may lead to improved outcomes [67]. 

Current inpatient and outpatient therapy sessions are not optimal, as compared to the above literature reports. Typical inpatient rehabilitation sessions in the United States last ~39 min/day for ~12 days poststroke [68]. Outpatient rehabilitation sessions in the United States last ~36 min/day with patients engaging in an average of 12 purposeful movements in an otherwise unstructured treatment session, continuing for a few weeks [69]. Advancements in technology applications in stroke rehabilitation make it possible to increase therapy time and the patient’s engagement. Robot-assisted training with videogaming appears to be an attractive approach for upper-limb recovery for inpatient stroke rehabilitation [70]. Home-based robot-assisted training or virtual-reality training make it feasible to supplement outpatient therapy sessions [71,72,73].

#### 3.2.4. Interventions to Address Complications

Along with continuous recovery, other motor impairments, such as abnormal synergy and spasticity, are likely to develop. Spasticity is present in up to 97% of stroke survivors with moderate to severe motor impairments [74]. Spasticity can interact with weakness, and worsen the motor functions (difficulty walking, reaching, grasping etc). For example, intended hand opening could result in hand closing due to involuntary activation associated with finger flexor spasticity [75]. In the acute phase, early detection and suppression of spasticity via botulinum toxin injections can prevent contracture development and maintain the range of motion of affected joints, while progress in motor recovery is not negatively affected [76]. In the chronic phase, appropriate and adequate management of ankle plantar flexor spasticity can correct ankle and foot joint abnormality and placement [77], and improve gait speed in ambulatory stroke survivors with spastic equinus foot [78].

#### 3.2.5. Combination of Interventions

It is of scientific rigor to control confounding factors and assess the effectiveness of an individual intervention. In real practice, combining different interventions and modalities is likely to yield better clinical outcomes, especially when interventions target different areas. For example, to improve hand function in stroke patients with spastic hemiplegia, a botulinum toxin injection was used to reduce finger flexor spasticity, and electrical stimulation to strengthen finger extensor muscles. In addition, the patient received intense therapy 1 h per day for 4 weeks [79]. Although it is known that medication alone (botulinum toxin therapy) [80] or electrical stimulation alone ([81,82] is not effective in improving the motor function of the hand in chronic stroke survivors, results from this study [79] demonstrated that different interventions could work synergistically to achieve optimal clinical results. This concept of combining interventions to better recovery after a stroke is supported by results from other studies, such as medication and tDCS [83], tDCS and robot-assisted training [84], mirror therapy and electrical stimulation [85], mirror therapy and non-invasive brain stimulation [86], Vagus nerve stimulation and intense therapies [87].

### 3.3. Maintenance of Motor Function

**Figure 3:**
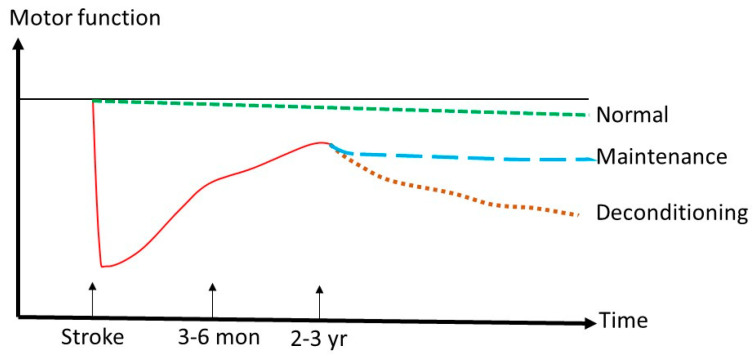
Schematic illustration of longitudinal view of recovery and maintenance of motor function after a stroke. Stroke survivors have a predicted curve of motor recovery (red line). With a programmatic approach, they may be able to maintain their motor function (blue line) as compared to age-matched counterparts (green) with a normal rate of decline. Many factors may lead to a faster functional decline (brown line).

There are heterogeneous reports in the literature on motor function after discharge from inpatient rehabilitation. Many studies have reported that the motor function of stroke survivors continues to improve up to 2~3 years post-stroke [20,88,89] (Figure 3). Stroke survivors with regular exercises can maintain motor functions and overall quality of life 4 years after the incident [90]. Others have reported functional decline over time since the discharge to home [89,91]. Meyer et al. reported that the functional and motor outcome at 5 years after a stroke is equivalent to the outcome at 2 months [92]. Some patient characteristics or clinical variables are associated with deterioration of the outcome several years after stroke rehabilitation, including higher age, stroke severity, concomitant chronic disorders, cognitive problems, and depression [93,94].

Some factors are modifiable and play an important role in the maintenance of motor functions in chronic stroke survivors. Even after stroke survivors return to living in the community, their levels of physical activity remain lower than their age-matched counterparts [95]. Community-dwelling stroke survivors spend the vast majority of their waking time sitting down [96]. A decreased physical activity level is associated with an increased risk of cardiovascular disease and diabetes [97]. Furthermore, the risk of malnutrition is high, especially in older stroke survivors [98,99]. Both a decline in mobility and malnutrition contribute to wasting of skeletal muscles, i.e., sarcopenia [24], which in turn negatively affects muscle strength and motor function [100]. Motivation and social participation as well as resources available to stroke survivors often generate synergy to maintain and improve their activities of daily living [88,101]. Collectively, programmatic approaches targeting exercise, nutrition and motivation can enable stroke survivors to build confidence to engage in self-managed practice routines and maintenance of motor function [102].

### 3.4. Family and Societal Support

Stroke recovery can be a long and challenging process for the family and society as well. The inability of stroke survivors to adequately perform basic activities imposes a significant burden on their caregivers, specifically informal caregivers who are not hired to provide caregiving services, usually family members [103,104]. At least one-third of caregivers reported having spent a moderate to a great deal of time assisting with nursing, personal care, walking, and transfer (e.g., from bed to a chair) tasks. More than two-thirds spent a moderate to a great deal of time providing emotional support, monitoring the stroke survivor’s progress, talking to health care professionals regarding the stroke survivor’s condition and treatment plan, providing transportation, helping with additional tasks at home and outside the home, and managing the stroke survivor’s finances and medical bills [104]. Furthermore, this study also found that the caregiver’s burden increased with the level of the stroke survivor’s disability [104]. Informal caring for stroke survivors is associated with humanistic costs including decrements in health-related quality of life. Another study reported caregivers had significant humanistic burdens such as depression and anxiety [105]. Overall, it has been reported that the quality of life of caregivers has been significantly affected [103]. The family’s ability to provide adequate caregiving services is important for stroke survivors to stay engaged in their rehabilitation program and to set achievable goals for themselves. A recent study revealed that pre-stroke socioeconomic status (SES) predicts upper-limb motor recovery after inpatient neurorehabilitation [106]. Higher pre-stroke SES is associated with more frequent use of outpatient therapy, and better access to resources, thus better long-term recovery after discharge from rehabilitation [106]. Understanding these factors is important to advocate policy changes to improve access to healthcare for all stroke survivors.

## 4. Concluding Remarks

Stroke recovery is a journey for stroke survivors. The journey starts with the stroke onset and may last for the rest of their life. Prediction of who will recover after a stroke and to what extent has been a constant focus in the field of rehabilitation. Assessment of initial impairments allows a reasonable prediction of biological spontaneous recovery at 3 to 6 months poststroke. Stroke survivors usually continue to improve their motor function for the first several years, but their motor function is likely to decline afterwards. A recovery model within the ICF theoretical framework is proposed here to identify opportunities and manage barriers to achieve maximum recovery and maintain motor function throughout the journey. The ICF recovery model involves health optimization, comprehensive rehabilitation, support from family and caregivers, and a commitment to a healthy lifestyle. Health conditions of individuals and medical and neurological complications can be optimally managed under the care of specialized physicians. Health optimization is particularly important in early recovery to minimize the interruption of and to facilitate participation in various therapies and therapeutic interventions. This could potentially change the trajectory of motor recovery. Applications of novel interventions can improve the motor functions and independent living of these individuals. A sufficient dose of appropriate interventions at the right time is critical for stroke motor rehabilitation. There is evidence of a critical period when stroke survivors are most plastic and sensitive to therapeutic interventions. However, there is currently insufficient evidence to recommend a minimum beneficial daily amount in clinical practice. It is important to highlight that combining interventions is likely to yield the best clinical outcomes. Caregivers, including family members, can assist and facilitate targeted therapeutic exercises for these individuals, through planned independent-living training. Furthermore, caregivers can help them comply with medical plans (medications, visits), and provide emotional support. Family support and adequate nutrition and a commitment to a healthy lifestyle are key to maintaining their motor function and independence in the later phase of motor recovery. This interdisciplinary care approach is likely to achieve their recovery potentials. The ICF recovery model is likely to provide a guidance through the journey to best achieve stroke recovery potentials. At the present time, the evidence of ICF usage in stroke rehabilitation is scarce [107,108]. Clinicians often face the burden of extracting the ICF codes. A recent report showed the applicability of Chat-GPT to extract ICF codes to assist clinical decision making [109].

## Data Availability

The datasets used to support the findings of this study are available from the corresponding author upon request.
